# Expanding Transposase Technology to Regulatory Noncoding RNAs for Stable Expression in CHO Cells

**DOI:** 10.1002/biot.70299

**Published:** 2026-08-23

**Authors:** Sonja Lochmueller, Linus Weiss, Kerstin Otte

**Affiliations:** ^1^ Ulm University Ulm Germany; ^2^ Institute for Applied Biotechnology, University of Applied Sciences Biberach Biberach Germany

**Keywords:** cell engineering, CHO, microRNA, random integration, transposase‐mediated integration

## Abstract

Stable expression of regulatory non‐coding RNAs enables targeted cell engineering in Chinese hamster ovary (CHO) cells but commonly relies on random integration, resulting in variable expression. Here, we establish a transposase‐mediated platform for stable microRNA (miRNA) expression in CHO cells and demonstrate the first application of a DNA transposon system for long‐term miRNA genome integration. Using miR‐3096b‐5p, we compared *p* *iggyBac*‐mediated integration with conventional random integration in antibody‐producing CHO cells. Transposase‐mediated integration enabled rapid generation of stable cell pools with higher fractions of GFP‐expressing cells. Both integration strategies supported comparable growth, viability, and antibody production in batch cultures, indicating that transposase‐mediated miRNA integration does not compromise bioprocess performance. At the molecular level, transposase‐mediated integration resulted in significantly higher transgene copy numbers and markedly increased miRNA expression, translating into stronger and more consistent regulation of the target genes *Fuk* and *Gmds*. By combining high integration efficiency and expression stability with the regulatory potential of miRNAs, this approach expands the applicability of transposase systems beyond protein‐coding transgenes and provides a robust platform for precise and durable post‐transcriptional gene regulation in CHO cell engineering.

## Graphical Abstract and Lay Summary

Compared with random integration, *piggyBac*‐mediated integration enhanced stable miRNA expression and target gene regulation in CHO cells without affecting bioprocess performance.

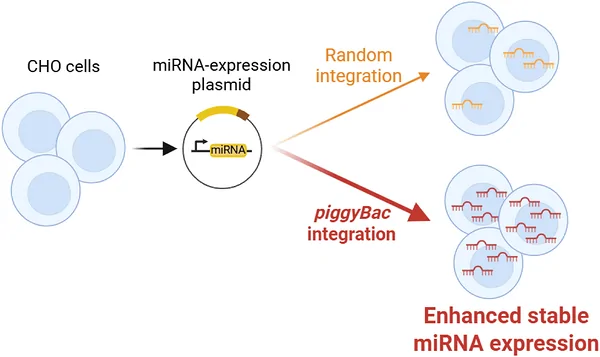

**Abbreviations**
CHO: chinese hamster ovary
ITRs: inverted terminal repeats
ncRNAs: Non‐coding RNAs
miRNAs: microRNAs
mAb: monoclonal antibody
PB: *piggyBac*
PB‐ITRs: *piggyBac* inverted terminal repeats
qPCR: quantitative PCR
cDNA: complementary DNA
HPLC: high‐performance liquid chromatography
SD: standard deviation
*Fuk*
: L‐fucose kinase
*Gmds*
: GDP‐mannose 4,6‐dehydratase
Ct: quantification cycle
CPM: counts per million
Log_2_(CPM): Log_2_‐transformed counts per million
*Gapdh*
: glyceraldehyde‐3‐Phosphate Dehydrogenase
*Qp*
: specific productivity

## 1 Introduction

Stable genomic integration of heterologous DNA sequences is a cornerstone of mammalian cell line engineering. Historically, this has been achieved by random plasmid integration, in which transfected DNA becomes incorporated into the host genome at unpredictable loci [1]. Although technically straightforward, random integration has several disadvantages. For example, integration events occur across the entire genome, resulting in highly heterogeneous cell pools characterized by variable expression levels, extensive screening for the identification of desired clonal cell lines, or reduced stability over time [2, 3, 4]. This makes random integration a poorly predictable approach that, despite its limitations, remained the prevailing standard for the establishment of stable expression pools for many years [5].

Barbara McClintock's discovery and the molecular characterization of transposable elements provide an alternative to this paradigm [6]. DNA transposases recognize specific inverted terminal repeats (ITRs) and catalyze the excision and reintegration of the DNA fragment flanked by ITRs via a cut‐and‐paste mechanism [7]. This can be used for biotechnological purposes, as virtually any DNA sequence can be placed between ITRs and stably integrated into the host genome by trans‐supply of the transposase [8]. Since integration does not occur at a predefined genomic locus, but transposases preferentially insert transgenes into actively transcribed and accessible genomic regions guided by specific sequence requirements, the process is referred to as semi‐targeted integration [9].

Compared to random plasmid integration, transposase‐mediated integration is markedly more efficient, occurs at higher copy numbers, and shows a strong tendency toward integration into transcriptionally active genomic regions, which results in more robust, predictable, and durable expression [10, 11, 12, 13, 14]. Consequently, transposase systems have emerged as a new gold standard for stable gene delivery in mammalian cells, with applications ranging from functional genomics to the generation of high‐yielding production cell lines for recombinant proteins and monoclonal antibodies (mAbs) [5, 15]. Until now, the use of transposon technology in mammalian systems has been limited to the transfer of protein‐coding transgenes. In contrast, regulatory noncoding RNAs (ncRNAs) have not yet been systematically investigated as cargo for transposase‐mediated integration. This represents a major gap, given that ncRNAs are key post‐transcriptional regulators of gene expression. Among these, microRNAs (miRNAs) have proven particularly valuable as tools for modifying eukaryotic cells. MiRNAs of about 22 nucleotides in length regulate gene expression by binding to mRNAs and thereby blocking translation or promoting mRNA degradation [16, 17]. Because they can bind imperfectly to the 3′ untranslated regions of many different mRNAs, miRNAs can influence whole networks of genes at the same time, which makes them attractive tools for cell line engineering [18]. In CHO cells, several studies have shown that overexpression of certain miRNAs can improve cell growth, product titer, specific productivity and might be used to modulate product quality, such as antibody glycosylation [19, 20, 21, 22, 23]. As a result, miRNA‐based engineering has moved beyond pure proof‐of‐concept and is now seen as a promising approach for developing next‐generation CHO production cell lines [21, 24]. More recently, miRNAs have also been suggested as candidates for engineering human cell lines for improved adeno‐associated virus production [25, 26, 27, 28]. This growing interest highlights the need for efficient and reliable integration methods that allow stable, long‐term miRNA expression.

In this study, we established transposase‐mediated integration as a platform for stable miRNA expression in CHO cells and directly compared its performance with conventional random integration. To our knowledge, this represents the first application of a DNA transposon system for the stable introduction of a functional miRNA expression cassette in this context. Unlike conventional protein‐coding transgenes, miRNAs require post‐transcriptional processing, maturation, and incorporation into the gene‐silencing machinery to exert their regulatory function [29, 30]. Therefore, it was unclear whether transposase‐mediated integration could support stable miRNA expression without impairing miRNA maturation or functional activity. By combining the high integration efficiency and expression stability of transposase systems with the regulatory potential of miRNAs, this approach expands the applicability of transposase‐based genome engineering beyond protein‐coding transgenes and provides a strategy for durable post‐transcriptional gene regulation in CHO cells.

## 2 Materials and Methods

### 2.1 Cell Culture

Maintenance cultures of mAb‐producing CHO cells were cultivated in TubeSpin Bioreactor 50 tubes (TPP Techno Plastic Products AG, Switzerland) using SFM4CHO medium (Cytiva, USA). Standard cultivation conditions were maintained at 37°C, 5% CO_2_, and 85% relative humidity, with orbital shaking at 140 rpm and a 25 mm orbit (Kuhner Shaker GmbH, Germany). Cell concentration and viability were routinely monitored using a CEDEX XS cell counter (Roche Diagnostics, Germany) in combination with a trypan blue exclusion assay (Thermo Fisher, Germany). For maintenance, cells were passaged every three days to a density of 5 × 10^5^ viable cells/ml.

### 2.2 Plasmids and Cloning

For miRNA overexpression, the commercially available BLOCK‐iT Pol II miR RNAi Expression Kit containing the pcDNA6.2‐GW/EmGFP‐miR plasmid (Thermo–Fisher Scientific, Germany) was used. The overexpression plasmid employed for random genomic integration has been described previously [22]. The pcDNA6.2‐GW/EmGFP‐miR‐negative control plasmid supplied with the kit served as a mock control. For insertion of p*iggyBac* inverted terminal repeats (PB‐ITRs), the pcDNA6.2‐GW/EmGFP‐miR‐negative control plasmid was PCR‐amplified using overhang primers containing *NotI* and *BspTI* restriction sites (Table S1). The 5′ PB‐ITR was amplified from pPB[Exp]‐CMV>PBase (VectorBuilder Inc., USA) using primers containing *BspTI* and *NotI* sites, while the 3′ PB‐ITR was amplified with primers carrying *Bst1107I* and *PciI* sites (Table S1). The 5′ PB‐ITR fragment was first inserted into pcDNA6.2‐GW/EmGFP‐miR‐neg. By restriction digestion and ligation, the resulting construct was subsequently used for insertion of the 3′ PB‐ITR. The tandem miRNA sequences, derived from the random integration plasmids, were then cloned into the pcDNA6.2‐GW/EmGFP‐PB‐ITR backbone via restriction digestion and ligation using *BamHI* and *XhoI*. All resulting plasmids were verified by Sanger sequencing to confirm correct insertion (Eurofins Genomics Europe Sequencing, Germany). The mature miR‐3096b‐5p sequence cloned into the expression plasmid was based on the published murine miRNA sequence reported by Schlossbauer et al. and is listed in Table S2.

### 2.3 Stable Transfection of miRNA Plasmids

CHO‐mAb cells were transfected using the Neon Transfection System (Thermo–Fisher Scientific, USA) according to the manufacturer's instructions. Briefly, 5 × 10^6^ cells with a viability greater than 95% were pelleted, resuspended in Buffer R, and mixed with a total of 7 µg circular plasmid DNA in a 100 µL Neon tip. For transposase‐mediated integration, the transposase plasmid (pRP[Exp]‐CMV>hyPBase, VectorBuilder Inc., USA) was added at 10% of the total DNA amount, while water served as a selection control. Electroporation was performed at 1650 V for 20 ms with a single pulse. Following transfection, cells were transferred into 5 mL of pre‐warmed medium and cultured in 6‐well plates (Greiner Bio‐One, Germany) at 37°C, 80% relative humidity, 5% CO_2_, and 140 rpm. Transfection efficiency was determined 48 h post‐transfection by measuring GFP fluorescence. Selection was initiated by adding 6 µg/mL blasticidin (InvivoGen, USA) to the culture medium, which was maintained throughout each passaging step until full recovery of cell viability. Cells were passaged every two to three days, and cell concentration, viability and GFP‐positive cells were monitored at each passage. After completion of selection, batch cultivations were performed in 30 mL PowerCHO medium (Lonza Group Ltd, Switzerland) for five days. Harvested cultures were used for subsequent analytical assays.

### 2.4 Flow Cytometry

The proportion of GFP‐positive cells was measured after transfection and during the selection phase using the MACSQuant Analyzer 10 (Miltenyi Biotec, Germany). GFP was measured at 525/50 nm using a 488 nm laser. Subsequent data analysis was performed with the MACSQuantify software.

### 2.5 Quantitative Reverse Transcription Polymerase Chain Reaction (qPCR)

Total RNA was isolated using the miRNeasy Mini Kit (Qiagen, Germany) according to the manufacturer's protocol. For target regulation analysis, complementary DNA (cDNA) was synthesized using the High‐Capacity cDNA Reverse Transcription Kit (Applied Biosystems, USA). Quantitative PCR (qPCR) was performed on a LightCycler 480 instrument (Roche, Switzerland) using CHO‐specific TaqMan (Thermo Fisher Scientific Inc., USA) gene expression assays for L‐fucose kinase (*Fuk*, Cg04505402_m1) and GDP‐mannose 4,6‐dehydratase (*Gmds*, Cg04422381_m1). Glyceraldehyde‐3‐Phosphate Dehydrogenase (*Gapdh*, Cg04424038_gH) served as the endogenous reference gene. Amplifications were carried out following the TaqMan Gene Expression Master Mix protocol (Applied Biosystems, USA).

For miRNA quantification, cDNA synthesis was performed using the miRCURY LNA RT Kit (#339340, Qiagen). qPCR was subsequently carried out on the same instrument using the miRCURY SYBR Green PCR Kit (Qiagen). Specific miRCURY LNA miRNA PCR primers were applied for miR‐3096b‐5p (custom synthesis based on the mature sequence) and for the endogenous control mmu‐U6‐snRNA (YP02119464), following the manufacturer's instructions. Data were analyzed using the 2^−ΔΔCt^ method.

For relative quantification of transgene copy numbers, genomic DNA was isolated using the Monarch Genomic DNA Purification Kit (New England Biolabs, Germany). Standard curves for each primer set were generated from serial dilutions of genomic DNA to determine amplification efficiencies. Gene copy numbers were calculated by comparing amplification of the *blasticidin* gene to the single‐copy endogenous control gene *Ptpnr2*, as previously described [31].

### 2.6 Gene Expression Analysis by RNA Sequencing

Gene expression analysis was conducted with RNA sequencing technology as described [32].

### 2.7 Protein A Chromatography

Antibody concentrations in culture supernatants from randomly and transposase‐transfected CHO‐mAb cells were determined by analytical protein A high‐performance liquid chromatography (HPLC). Supernatants were filtered through 0.2 µm AcroPrep Advance 96 filter plates (Pall Corporation, USA) and analyzed using an UltiMate 3000 HPLC system (Thermo Fisher Scientific Inc., USA) equipped with a POROS A 20 µm Protein A column (Thermo Fisher Scientific Inc.). Phosphate buffer (pH 7.5) was used for column equilibration, and phosphate buffer at pH 2.5 for elution. Antibody concentrations were quantified by measuring UV absorbance at 280 nm.

### 2.8 Statistical Analysis

Statistical analyses were performed using GraphPad Prism (Version 8.0.1; GraphPad Software, San Diego, CA, USA). For detailed information on statistical test applied, please refer to the respective figure legends. All data sets are given as (biological/technical) triplicates as mean ± SD.

## 3 Results

### 3.1 Design of a *p* *iggyBac*‐based miRNA Expression System

**FIGURE 1:**
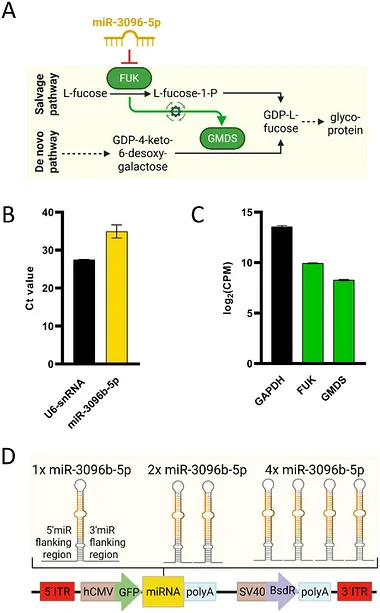
Expression profiling and construct design for the fucose biosynthesis pathway modulating miR‐3096b‐5p. (A) Schematic representation of the fucose biosynthesis pathway illustrating the regulatory interplay between L‐fucose kinase (*Fuk*) and GDP‐mannose 4,6‐dehydratase (*Gmds*). Downregulation of *Fuk‐*mRNA by miR‐3096b‐5p reduces the availability of GDP‐L‐fucose, which acts as a feedback activator of *Gmds*, thereby indirectly releasing *Gmds* from inhibition and promoting its upregulation. Created with BioRender.com. (B) Basal expression analysis of miR‐3096b‐5p and (C) transcript levels of its predicted target genes in CHO cells. Shown are the quantification cycle threshold (Ct) values for miR‐3096b‐5p and the reference control mmu‐U6‐snRNA determined by qPCR, as well as the transcript levels of Glyceraldehyde‐3‐Phosphate Dehydrogenase (*Gapdh*), *Fuk* and *Gmds*. Gene expression data were normalized as counts per million (CPM) and log_2_‐transformed (log_2_(CPM)). n = 3 biological replicates, mean ± standard deviation. (D) Schematic design of the miRNA expression constructs based on the modified p*iggyBac* plasmid backbone. Mature miR‐3096b‐5p sequences were inserted into miR‐155 flanking regions located within the 3′UTR of a GFP reporter gene. Constructs containing one, two, or four tandem copies of miR‐3096b‐5p were generated to enable tunable regulatory strength. Created with BioRender.com.

While transposase‐mediated integration has so far been limited to protein‐coding genes, its extension to regulatory ncRNAs in CHO antibody‐producing cells offers a novel and versatile strategy for stable expression and cell engineering. For the establishment of a transposase‐based integration system, miR‐3096b‐5p was selected as a model ncRNA, as we previously showed that it targets L‐fucose kinase (*Fuk*) and GDP‐mannose 4,6‐dehydratase (*Gmds*) [33]. While miR‐3096b‐5p was shown to directly downregulate *Fuk*‐mRNA expression in the *salvage* pathway for protein fucosylation, a compensatory feedback upregulation of *Gmds*‐mRNA expression in the *de novo* pathway was observed (Figure 1A). This reciprocal regulation renders *Fuk* and *Gmds* particularly informative target genes to study miRNA effectiveness and functionality. To verify the expression of all components in our model system, we first assessed the basal levels of miR‐3096b‐5p in CHO‐mAb cells by qPCR, which revealed low expression (Ct >30), facilitating sensitive detection of integration events (Figure 1B). Furthermore, transcriptomic analysis showed that the miR‐3096b‐5p target genes *Fuk* and *Gmds* were expressed at intermediate levels, allowing for robust monitoring of miRNA‐mediated gene regulation (Figure 1C).

To create a miRNA expression plasmid for stable transposase‐mediated integration into the CHO genome, we adapted a previously published miRNA expression plasmid by incorporating PB‐ITRs (Figure 1D) [22]. The resulting construct was used to insert 1, 2, or 4 copies of miR‐3096b‐5p sequences into the 3′UTR of a GFP reporter gene, thereby coupling miRNA expression to a fluorescent readout (Figure 1D). This allowed simultaneous assessment of two distinct molecular outputs, namely protein‐coding expression through GFP and post‐transcriptional regulatory activity through the miRNA units. The 1x, 2x, and 4x constructs were designed to mimic the clustered organization of endogenous miRNAs, which are often transcribed as long RNA polymerase II‐driven primary transcripts [34, 35]. In addition, a mock plasmid without targeting miRNA sequence was constructed as a control [33].

### 3.2 Transposase‐Mediated Genome Integration of miRNAs is Fast and Efficient

As random integration of miRNAs is currently the gold standard for CHO cell engineering, the above newly established transposase‐based system was tested in direct comparison. Therefore, CHO antibody‐producing cells were transfected with either the control or 1x, 2x, or 4x‐miRNA‐containing constructs alone, or each construct together with the hyperactive *p* *iggyBac* (PB) transposase‐expression plasmid. To achieve stable incorporation of the miR‐3096b‐5p cassettes, antibiotic selection pressure was applied in both cases and GFP expression and cell viability were monitored. Selection continued until the viability and the proportion of GFP‐positive cells reached a stable plateau, indicating an enrichment of cells with stably maintained expression cassettes and minimizing the contribution of transient plasmid expression.

**FIGURE 2:**
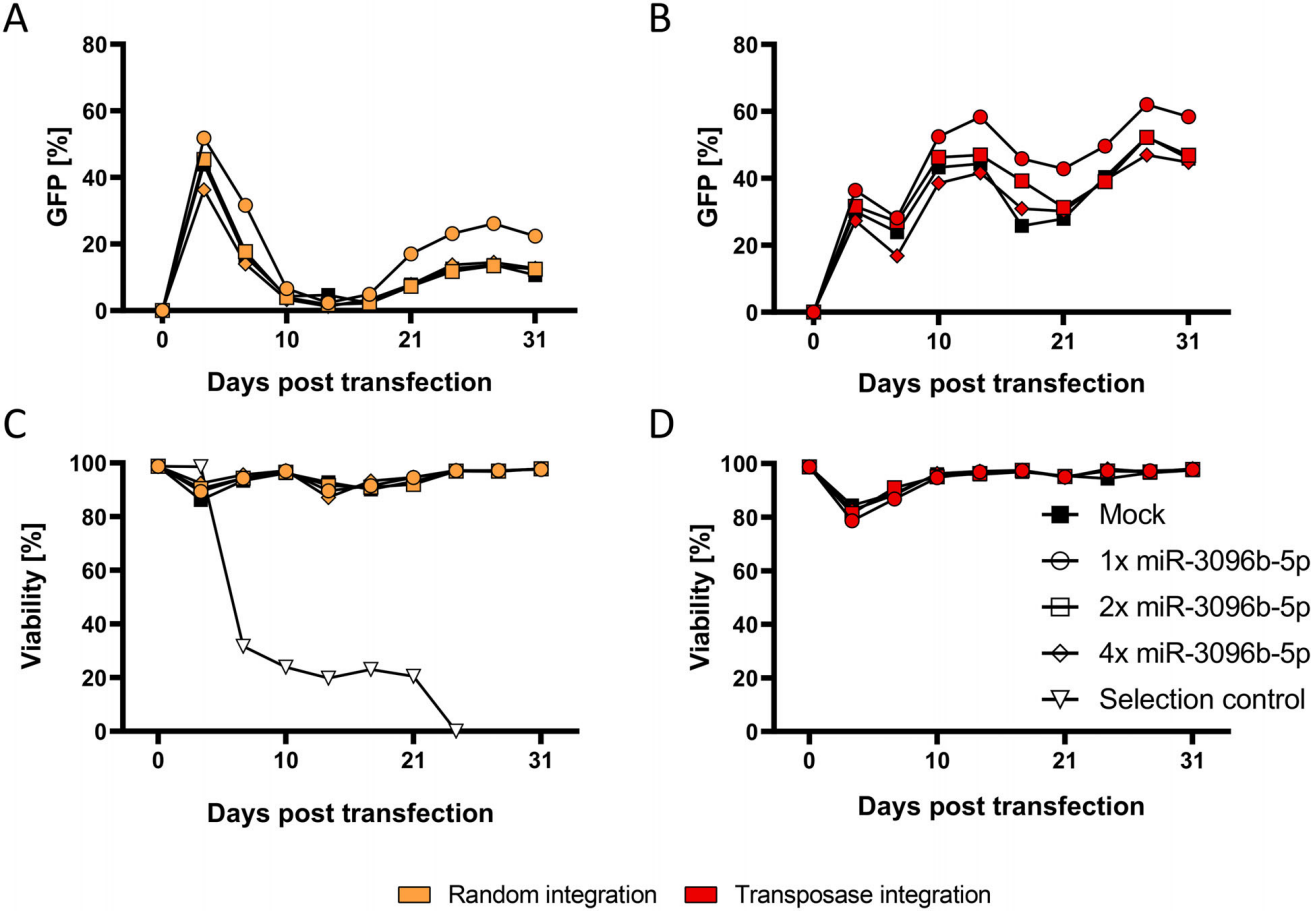
Establishment of stable miRNA‐expressing CHO cell pools via random and transposase‐mediated integration. (A) Establishment of stable miRNA‐expressing cell pools via random plasmid integration. CHO‐monoclonal antibody (mAb)‐producing cells were transfected with constructs containing one, two, or four copies of miR‐3096b‐5p, or a non‐targeting miRNA (mock). GFP‐positive cell populations and cell viability were monitored over 31 days of antibiotic selection. (B) Generation of stable miRNA‐expressing cell pools through transposase‐mediated integration. CHO‐mAb‐producing cells were co‐transfected with miR‐3096b‐5p expression constructs containing one, two, or four tandem copies together with a hyperactive p*iggyBac* transposase‐expressing plasmid. Cells transfected with a nontargeting miRNA construct served as mock control. GFP fluorescence and cell viability were measured throughout a 31‐day antibiotic selection period. (C) Measurement of viability during selection for stable miRNA‐expressing CHO cell pools for random plasmid integration. Cells transfected with water served as a selection control. Viability was determined alongside the GFP+ cells over a period of 31 days during antibiotic selection. (D) Measurement of viability during selection for stable miRNA‐expressing CHO cell pools for transposase‐mediated integration. Viability was determined alongside the GFP+ cells over a period of 31 days during antibiotic selection.

For the random integration approach without transposase‐plasmid, GFP‐positive cells gradually emerged under selective pressure, indicating enrichment of cells successfully integrating the miRNA‐containing transgene (Figure 2A). GFP levels stabilized by day 31 for miRNA‐expressing constructs, reaching 20 % GFP‐positive cells on average, with the mock control reaching similar levels of GFP expression. In contrast to random integration, PB‐mediated transposition led to an earlier increase in GFP expression, which finally reached a strong and stable fluorescence of 50%–60% GFP‐positive cells across the different miRNA constructs by day 31 (Figure 2B). This confirms efficient and durable integration, which was also seen for the controls.

The random (Figure 2C) and transposase‐mediated integration approaches (Figure 2D) caused a notable transient decline in cell viability immediately after electroporation. After this initial decline, the cells recovered and maintained consistently high viability throughout the selection period for all constructs. The only exception was the selection control (Figure 2C). These data demonstrate that, compared with random integration, PB‐mediated transposition supports effective selection and preserves cell fitness. Although final stabilization was assessed after 31 days for both approaches, PB‐mediated transposition led to a greater and earlier accumulation of GFP‐positive cells, thereby accelerating the establishment of high‐level transgene expression during selection.

### 3.3 Comparable Bioprocess Performance of Random and Transposase‐Mediated miRNA Integration

**FIGURE 3:**
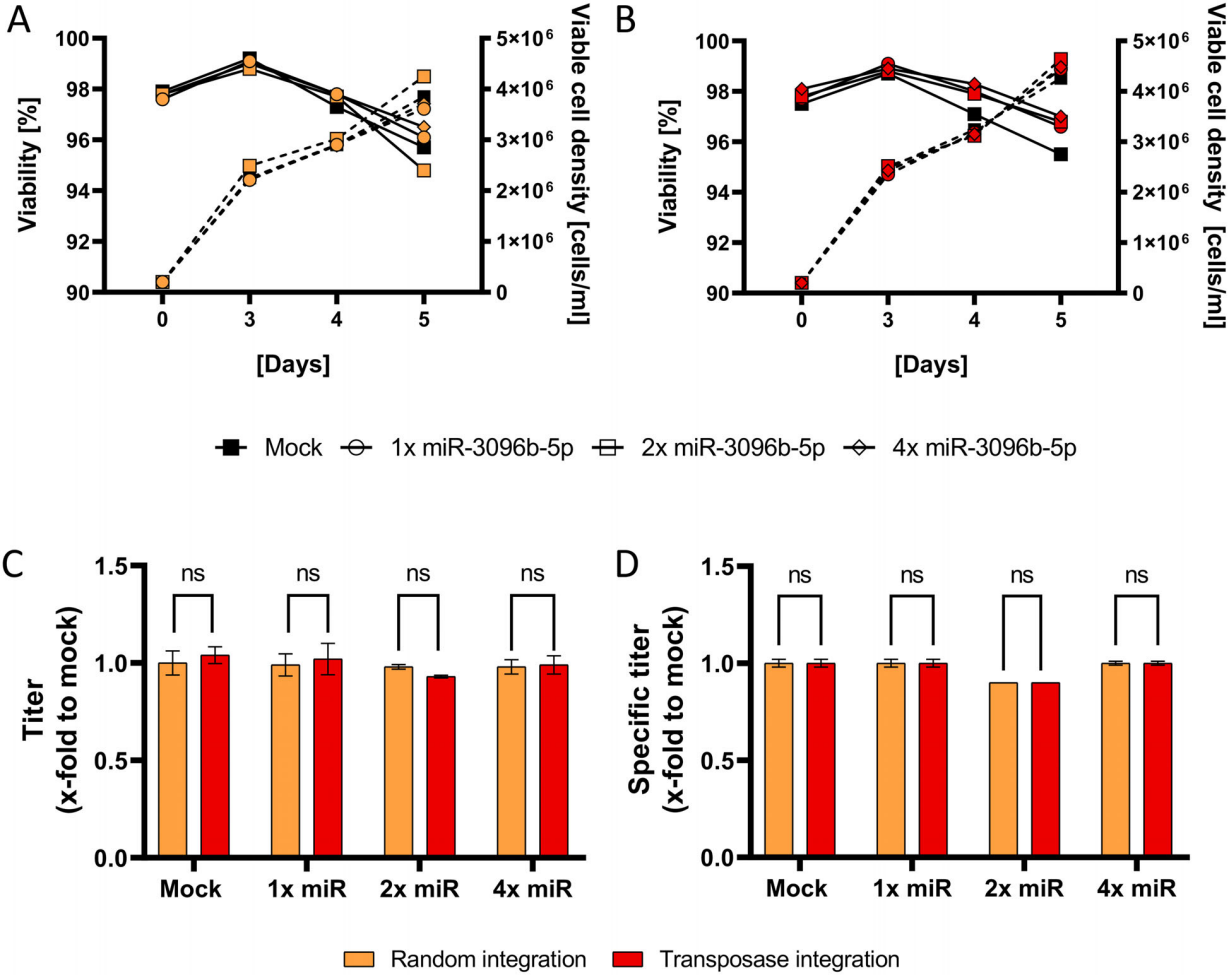
Effects of stable miRNA integration on CHO bioprocess parameters. (A) Batch cultivation of CHO‐monoclonal antibody (mAb)‐producing cell pools stably expressing miR‐3096b‐5p via random integration. Cell viability and viable cell density were monitored over a five‐day cultivation period. (B) Batch cultivation of CHO‐mAb‐producing cell pools stably expressing miR‐3096b‐5p via transposase‐mediated integration. Cell viability and viable cell density were monitored over a five‐day cultivation period. (C) Total antibody titers relative to mock and (D) specific productivity (Qp) after batch cultivation were assayed via analytical protein A chromatography. Significance was tested by ordinary one‐way ANOVA (ns: *p* > 0.05) *n* = 3 technical triplicates, mean ± standard deviation.

To evaluate the impact of random and PB‐mediated miRNA genome integration on CHO bioprocess performance, stable cell pools were subjected to batch cultivation over a five‐day period. Both integration strategies exhibited comparable growth profiles across all constructs, maintaining high viability and supporting robust cell proliferation (Figure 3A,B). In addition, titer analysis at the end of cultivation revealed that the miRNA integration method had no significant impact on production performance (Figure 3C). The same was observed for specific productivity (Qp), which remained consistent across all tested conditions (Figure 3D).

These findings indicate that cell growth, viability, and productivity in CHO cells remain unaffected by the genome integration strategy used for the miR‐3096b‐5p expression constructs.

### 3.4 Transposase System Enables Stronger miRNA Expression and Target Regulation

**FIGURE 4:**
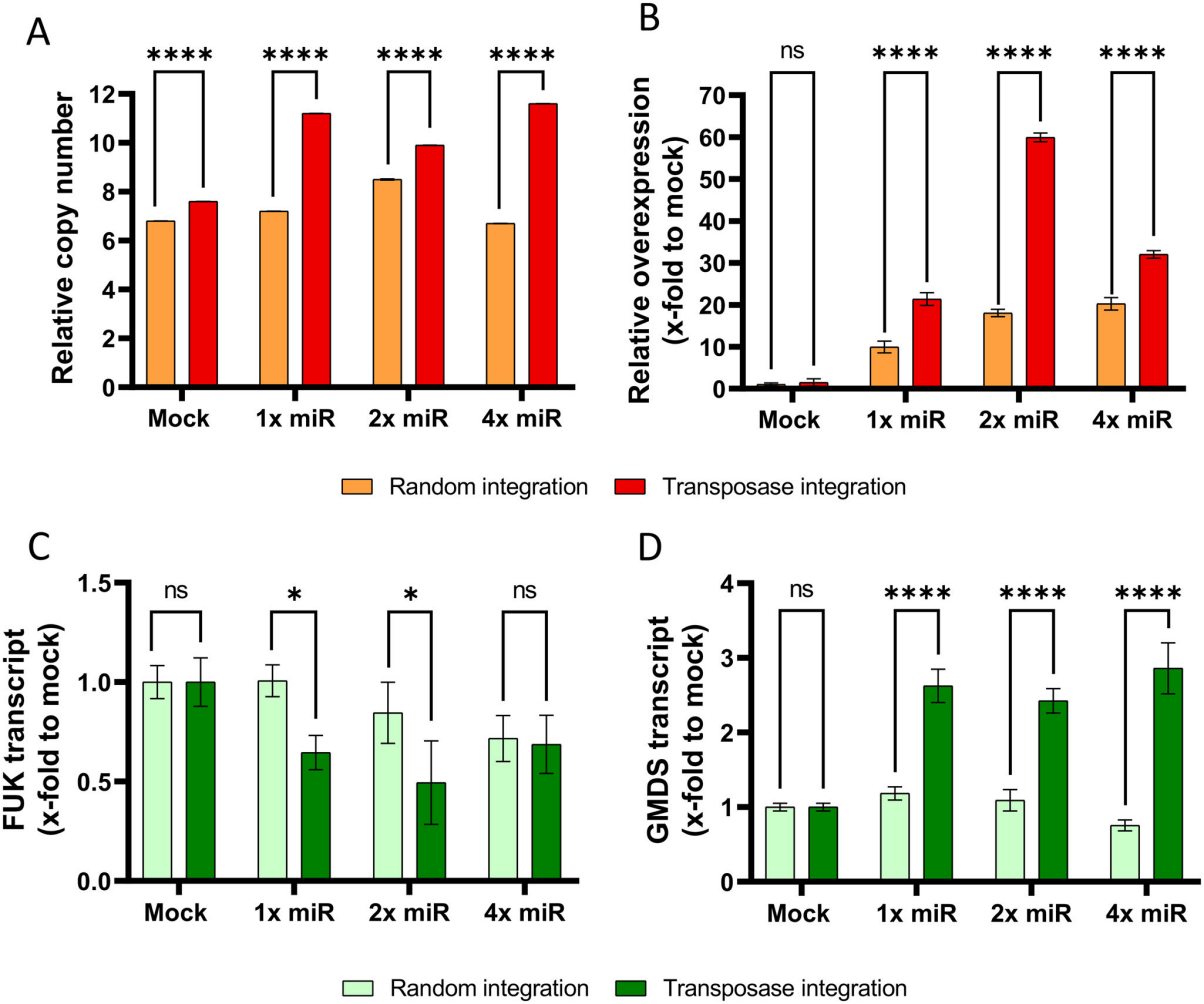
Quantification of transgene cassette number, expression, and target gene regulation after random and transposase‐mediated integration. (A) Transgene cassette copy number determination by qPCR for constructs containing one, two, or four copies of miR‐3096b‐5p in CHO monoclonal antibody (mAb)‐producing cell pools generated via random or transposase‐mediated integration. qPCR data are presented as calculated x‐fold change normalized to the *Ptpnr2* gene. (B) Quantification of mature miRNA‐3096b‐5p in stably overexpressing CHO‐mAb‐producing cell pools either integrated by random or transposase‐mediated integration. qPCR data are presented as calculated x‐fold change normalized to mmu‐U6‐snRNA housekeeping control and relative to mock. (C) Regulation of target genes L‐fucose kinase (*Fuk*) for miR‐3096b‐5p expressing CHO‐mAb producer pools assayed via qPCR. Data are presented as calculated fold change normalized to Glyceraldehyde‐3‐Phosphate Dehydrogenase (*Gapdh*) housekeeping control and relative to mock. (D) Quantification of GDP‐mannose 4,6‐dehydratase (*Gmds*) transcript in miR‐3096b‐5p‐expressing CHO‐mAb producer pools. Data are presented as calculated x‐fold change normalized to *Gapdh* housekeeping control and relative to mock. Significance was tested by ordinary two‐way ANOVA (**** = *p* ≤ 0.0001; ***: *p* ≤ 0.001; **: *p* ≤ 0.01; *: *p* ≤ 0.05; not significant (ns): *p* > 0.05; *n* = 3 technical triplicates, mean ± standard deviation).

To further characterize the applied genome integration strategy, transgene copy numbers of both systems were compared. qPCR‐based analysis revealed that transposase‐mediated integration resulted in significantly higher transgene copy numbers, with 11.2, 9.9, and 11.6 copies for the 1x, 2x, and 4x constructs, respectively, compared to random integration, which yielded only 7.2, 8.5, and 6.7 copies (Figure 4A). In addition, miRNA overexpression was markedly higher after PB‐mediated integration, reaching up to 20‐, 60‐, and 33‐fold increases for the 1x, 2x, and 4x constructs, respectively, compared to only 11‐, 17‐, and 22‐fold after random integration (Figure 4B).

As *Fuk* is a previously reported target gene of miR‐3096b‐5p (Figure 1A), we analyzed its expression in all stable miR‐3096b‐5p cell pools. Here, PB‐mediated integration demonstrated a greater regulatory capacity than random integration, where *Fuk* expression was reduced to 0.64‐fold, 0.49‐fold, and 0.68‐fold for 1x, 2x, and 4x expression constructs (Figure 4C). In contrast, random integration showed no regulation for the 1x construct and only moderate downregulation to 0.84‐fold and 0.71‐fold for 2x and 4x expression constructs. These data point towards a greater regulatory capacity for transposase‐integrated miRNAs.

Since strong downregulation of *Fuk* can limit the pathway product GDP‐L‐fucose, a compensatory feedback upregulation of *Gmds*‐mRNA expression was previously reported (Figure 1A), [33]. Therefore, the expression of *Gmds* was also analyzed after stable miRNA integration. While PB‐mediated integration led to a marked upregulation of *Gmds* expression, with 2.62‐fold, 2.42‐fold, and 2.86‐fold increases for 1x, 2x, and 4x constructs, respectively, random integration showed no detectable regulation across all constructs.

In conclusion, transposase‐mediated integration enabled a more efficient and functionally robust mode of miRNA expression and may represent a novel strategy for stable genome integration of ncRNAs in bioengineering applications.

## 4 Discussion

While transposon systems like PB have been extensively validated for stable expression of protein‐coding transgenes in CHO cells [12, 36, 37], their use for ncRNAs remains largely unexplored. Stable miRNA overexpression has emerged as a versatile strategy to modulate proliferation, apoptosis, metabolism, and product quality traits in CHO cells, with several studies showing that miRNAs can enhance recombinant protein production or adjust glycosylation profiles [20, 22, 33, 38]. In line with these reports, miR‐3096b‐5p was selected as a model miRNA previously associated with the regulation of the fucosylation‐related genes *Fuk* and *Gmds* [22, 33]. In the present study, this model system was applied to evaluate stable miRNA cassette integration, miRNA overexpression, and target gene regulation.

First, PB‐ITRs were directly adapted to a miRNA expression platform, enabling stable integration of miRNA into CHO mAb‐producing cells. This resulted in improved selection, as demonstrated by higher GFP‐positive proportions compared to random integration. While GFP expression served as a reporter for selection, miRNA overexpression and target gene regulation demonstrated that the miRNA component remained functionally active. Together, these findings extend previous reports of efficient PB‐mediated integration and stable expression of protein‐coding transgenes in CHO cells by demonstrating that the system also supports functional miRNA expression cassettes [13, 14]. DNA transposases recognize the terminal repeat sequences flanking the cargo, allowing heterologous expression cassettes to be mobilized as long as the required terminal elements remain intact. Natural non‐autonomous transposons illustrate this principle by retaining these terminal sequences while relying on a compatible transposase supplied in trans [15, 39].

Despite clear differences in integration dynamics, both random and transposase‐mediated miRNA integration yielded comparable batch culture performance over five days, with similar viable cell density profiles and high cell viabilities. Total antibody titers and specific productivity were also indistinguishable between the two strategies and across all constructs, indicating that implementation of the transposase system did not negatively affect recombinant protein production under the tested batch conditions. This equivalence in growth and productivity underscores that the choice of integration technology primarily impacts genetic and regulatory attributes rather than core bioprocess performance under the conditions tested. Consequently, transposase‐based miRNA engineering appears compatible with the existing CHO production platform under the batch conditions tested.

Additionally, transposase‐mediated integration outperformed random integration across multiple metrics, including higher transgene copies, stronger miRNA induction, and more robust target regulation. These advantages align with previous PB studies showing higher copy numbers and improved expression of protein‐coding transgenes in CHO cells compared with random integration [10, 12, 40]. Importantly, copy number determination reflected integrated plasmid or transposon copies rather than individual miRNA copies. Thus, similar measured copy numbers may correspond to different numbers of miRNA copies depending on whether the integrated construct contained 1, 2, or 4 miRNA copies. This is particularly relevant for the 1x random integration pool, which showed no detectable *Fuk* downregulation despite copy numbers comparable to the 2x and 4x pools. Previous studies have demonstrated stable miRNA expression and target gene regulation in other cell lines [22, 33]. The higher miRNA expression encoded by the 2x and 4x constructs may therefore have exceeded the threshold required for detectable target regulation, whereas the 1x construct may have remained below this level.

However, mature miRNA output is not expected to scale linearly with miRNA copies. Previous studies have shown that miRNA clustering can facilitate processing for certain miRNAs, while precursor‐to‐mature miRNA conversion may also become limiting in a sequence‐ and structure‐dependent manner [34, 41, 42]. In line with this, the 4x miRNA pool did not result in higher mature miRNA levels than the 2x construct after PB‐mediated integration, despite containing more miRNA copies. Instead, mature miRNA expression was higher in the 2x condition, suggesting that the 4x arrangement may have reduced processing efficiency or exceeded the optimal precursor architecture for this specific miRNA cassette. The absence of the same pattern after random integration might reflect the stronger influence of integration‐site‐dependent variability in expression in randomly integrated pools. Random integration can be influenced by genomic position, local chromatin context, tandem repeat formation, and silencing [5, 13, 43]. In contrast, PB‐mediated integration has been associated with transcriptionally active genomic regions and more stable expression behavior, which is consistent with the stronger miRNA induction and more robust regulation of *Fuk* and *Gmds* observed in this study [12, 13, 14].

Although the present study demonstrates the suitability of PB‐mediated integration for stable miRNA cassette delivery in an established CHO mAb‐producing cell line, further characterization of the genomic integration landscape will be important. Genome‐wide insertion site profiling would provide information on the location of the integration sites, chromatin state, and potential insertional effects. Moreover, the use of transposase systems in established production cell lines requires consideration of cassette stability during sequential engineering. Direct remobilization of the existing mAb expression cassette would only be expected if this cassette were flanked by compatible transposon ITRs. In our study, the cell line already contained the antibody expression cassette due to random integration prior to miRNA integration. Therefore, no transposase‐mediated cross‐mobilization of the cassette was expected, which is also supported by the unchanged antibody production observed after PB‐mediated miRNA cassette integration. A previous study has shown that PB can be used for simultaneous stable multigene expression in CHO cell pools [44]. However, in iterative engineering strategies, repeated expression of the same transposase could theoretically remobilize previously integrated cassettes flanked by transposons.

The transposase‐mediated miRNA integration platform might not be limited to CHO cell engineering and could find broad applicability beyond classical antibody production. In particular, cell‐based production of viral vectors has gained substantial importance in recent years. Emerging evidence highlights the critical role of miRNAs in optimizing producer cell lines such as HEK293 cells, where targeted miRNA modulation has been shown to improve viral vector yield and quality [25, 26, 27]. While these studies underscore the potential of miRNAs as powerful levers for enhancing AAV production, they also reveal a clear need for reliable and stable genome integration strategies to ensure consistent and durable miRNA overexpression.

Owing to the species‐independent mechanism of the PB‐transposase system, the platform presented here is likely to be directly transferable to HEK cells without the need for further adaptation. Collectively, this work substantially expands the functional scope of transposase‐based genome engineering and positions miRNA engineering as a precise and versatile tool for bioprocess optimization.

## Author Contributions

**Sonja Lochmueller**: investigation, data curation, writing – original draft, visualization. **Linus Weiss**: data curation, writing – review and editing. **Kerstin Otte**: writing – review and editing, supervision, project administration.

## Use of Generative AI and AI‐Assisted Technologies in the Writing Process

During the preparation of this work, the authors used ChatGPT 4o in a limited manner to improve the readability and language of the manuscript. The authors reviewed and edited the content and take full responsibility for the content of the published article.

## Funding

This work was supported by the Federal Ministry of Education and Research (BMBF), Germany (Grant number: 13FH036KB2).

## Conflicts of Interest

The authors declare no conflicts of interest.

## Supporting information




**Supporting File 1**: biot70299‐sup‐0001‐TableS1.docx.

**Supporting File 2**: biot70299‐sup‐0002‐TableS2.docx.

## Data Availability

The data that support the findings of this study are available in the supplementary material of this article.
