# Supplementary material for: Expanding Transposase Technology to Regulatory Noncoding RNAs for Stable Expression in CHO Cells
Source: Biotechnol J. 2026 Aug 23;21(8):e70299. doi: 10.1002/biot.70299 (PMC13501212; doi:10.1002/biot.70299)
Supplement: Supplementary file 1 [file BIOT-21-e70299-s001.docx]

**Supplement Table 1**

| **Primer** | **Sequence 5‘ 🡪 3‘** |
| --- | --- |
| 5′ PB-ITR fw (*BspTI*) | ATATCTTAAGGGTCGAGTAAAGCGCAAATCTT |
| 5′ PB-ITR rev (*NotI*) | ATATGCGGCCGCATGAACTAATGACCCCGTAATTGA |
| 3′ PB-ITR fw (*Bst1107I*) | ACAACATGACTGTTTTTAAAGTACAAA |
| 3′ PB-ITR rev (*PciI*) | ATATACATGTTTCACGACAGCAGGCTGAAT |
