# Supplementary material for: Expanding Transposase Technology to Regulatory Noncoding RNAs for Stable Expression in CHO Cells
Source: Biotechnol J. 2026 Aug 23;21(8):e70299. doi: 10.1002/biot.70299 (PMC13501212; doi:10.1002/biot.70299)
Supplement: Supplementary file 2 [file BIOT-21-e70299-s002.docx]

**Supplement Table 2**

| **miRNA** | **Sequence 5‘ 🡪 3‘** |
| --- | --- |
| miR-3096b-5p | GGCCAAGGAUGAGAACUCUAA |
